# Systemic Colonization by *Metarhizium robertsii* Enhances Cover Crop Growth

**DOI:** 10.3390/jof6020064

**Published:** 2020-05-17

**Authors:** Imtiaz Ahmad, María del Mar Jiménez-Gasco, Dawn S. Luthe, Mary E. Barbercheck

**Affiliations:** 1 Department of Entomology, The Pennsylvania State University, University Park, PA 16802, USA; meb34@psu.edu; 2 Department of Plant Pathology and Environmental Microbiology, The Pennsylvania State University, University Park, PA 16802, USA; mxj22@psu.edu; 3 Department of Plant Science, The Pennsylvania State University, University Park, PA 16802, USA; dsluthe@icloud.com

**Keywords:** cover crops, endophytes, fungal entomopathogens, *Metarhizium robertsii*, plant growth promotion, plant–microbe interactions

## Abstract

Fungi in the genus *Metarhizium* (Hypocreales: Clavicipitaceae) are insect pathogens that can establish as endophytes and can benefit their host plant. In field experiments, we observed a positive correlation between the prevalence of *M. robertsii* and legume cover crops, and a negative relationship with brassicaceous cover crops and with increasing proportion of cereal rye in mixtures. Here, we report the effects of endophytic *M. robertsii* on three cover crop species under greenhouse conditions. We inoculated seeds of Austrian winter pea (*Pisum sativum* L., AWP), cereal rye (*Secale cereale* L.), and winter canola (*Brassica napus* L.) with conidia of *M. robertsii* to assess the effects of endophytic colonization on cover crop growth. We recovered *M. robertsii* from 59%, 46%, and 39% of seed-inoculated AWP, cereal rye, and canola plants, respectively. Endophytic *M. robertsii* significantly increased height and above-ground biomass of AWP and cereal rye but did not affect chlorophyll content of any of the cover crop species. Among inoculated plants from which we recovered *M. robertsii*, above-ground biomass of AWP was positively correlated with the proportion of colonized root but not leaf tissue sections. Our results suggest that winter cover crops may help to conserve *Metarhizium* spp. in annual cropping systems.

## 1. Introduction

Several hypocrealean entomopathogenic fungi (EPF) commonly occur in soil [1] and in addition to infecting insects directly, can exist as rhizosphere colonizers and endophytes that provide multiple benefits in agroecosystems [2,3]. These benefits include plant growth promotion through nutrient transfers [4,5,6], plant disease suppression [7,8,9], and insect growth suppression [10,11,12]. Entomopathogenic fungi in the genus *Metarhizium* (Metschnikoff) Sorokin (Order Hypocreales: Family Clavicipitaceae) have a broad arthropod host range and are well-adapted to soil in agricultural systems [13,14,15]. Multiple species of *Metarhizium* are able to colonize the roots of many plant species, including switchgrass, haricot bean, tomato, wheat, and soybean [6,16,17,18,19,20]. Colonization of common bean by *Metarhizium robertsii* occurred through a prolonged rhizoplane colonization phase followed by transient low-level colonization of roots that persisted throughout the plant life cycle [21].

Plant growth promoting effects associated with endophytic colonization have been observed for multiple species of *Metarhizium* in tomato [18], maize [10,22], soybean [23], potato [24], cassava [19] and sweet pepper [25]. Application of *M. anisopliae* promoted the early development of peanut roots [26]. Inoculation with *M. robertsii* promoted lateral root growth and development of root hairs *Arabidopsis thaliana* seedlings in part through an auxin-dependent mechanism [27]. Soil drenching with *M. brunneum* resulted in an increase in sweet pepper growth parameters at 7- and 35-days post-inoculation [28]. Maize seeds inoculation with *M. anisopliae* was associated with increased stand density, and stalk and foliage fresh weight [29]. The authors attributed these effects to wireworm control and suggested that seed inoculation with *M. anisopliae* may be a novel method to increase stand density and yield of maize.

Rhizosphere-associated microbial communities are strongly influenced by plant species and soil characteristics in an agroecosystem [30,31]. Diverse plant species provide different species-specific resources such as metabolites, root exudates and other beneficial rhizosphere-associated compounds [32,33]. Plant species can also influence the occurrence of *Metarhizium* spp. [34]. For example, Wyrebek et al. [35] reported specificity of *Metarhizium* spp. in the rhizosphere and tissues of grasses. However, others have found no specificity in *M. robertsii*-plant associations [36].

Winter cover crops are grown between summer cash crops for benefits to soil and plant health. Winter cover crops provide benefits including, but not limited to, soil conservation, nutrient supply and retention, pest suppression and increased cash crop yield [37,38,39,40,41]. Growers are interested in using multi-species cover crop mixtures to provide a wide range of short- and long-term ecosystem services [42]. In an organically-managed cover crop–soybean–cover crop–maize rotation, *M. anisopliae* detection was greater in a cover crop mixture of timothy and red clover compared with a cereal rye and hairy vetch mixture [43]. In a field experiment to determine the effects of winter cover crop diversity in an annual cropping system on a variety of ecosystems services, detection of *M. robertsii* was lower in treatments with brassica cover crops as compared to those with legume cover crops [44]. Survival of conidia of formulated *M. anisopliae* applied to cereal rye and oat cover crops in a sugarbeet production system was greater in oat than in cereal rye, and the authors suggested that allelopathic compounds from cereal rye may have suppressed *M. anisopliae* [45].

Relatively fewer studies have reported the use of cover crops for the conservation of entomopathogens for biological control [13,46]. In this study, our goal was to determine the relative ability of *M. robertsii* isolated from soil from a field experiment [42] to colonize commonly-grown winter cover crop species. We tested the effects of *M. robertsii* inoculation of seeds of three cover crop species in three plant functional groups that were associated with different levels of detection of *M. robertsii* in the field: Austrian winter pea (*Pisum sativum* L. ‘Arvika’), cereal rye (*Secale cereale* L. ‘Aroostook’), and winter canola (*Brassica napus* L. ‘Wichita’) [44]. The specific objectives of this study were to assess: (1) the degree of endophytic colonization of plants by *M. robertsii*; (2) the tissue location of endophytic colonization of *M. robertsii* in cover crops grown from inoculated seeds; and (3) the effects of endophytic colonization by *M. robertsii* on cover crop growth. Based on our previous field studies [44], we hypothesized that: (1) colonization of Austrian winter pea would be greater than in cereal rye, and that colonization of canola would be lowest; (2) colonization of roots would be greater than that of foliage; and (3) growth promoting effects would be positively related to the intensity of colonization as determined by number of tissue sections colonized.

## 2. Materials and Methods

### 2.1. Fungal Inoculum

We used an isolate of *M. robertsii* J. F. Bischoff, Rehner and Humber originally collected from an annual agronomic cropping system experiment [42]. The field site was located at the Russell E. Larson Research and Education Center at Rock Springs, Pennsylvania, USA (43′40° N, 55′77° W, 350 m elevation) and managed in accordance with the USDA National Organic Standards [47], with no synthetic pesticide or fertilizer application [44]. We obtained the isolate of *M. robertsii* used in this experiment by sentinel insect baiting with larvae of *Galleria mellonella* of soil samples from an experimental plot containing winter canola (*Brassica napus* L. ‘Wichita’) [48]. We obtained pure cultures of this *M. robertsii* isolate from sporulating cadavers by culturing on dodine-free semi-selective CTC medium [49]. We confirmed the identity of fungi recovered from inoculated plants in the current experiment as *M. robertsii* by morphological and molecular characteristics [50,51]. We stored conidia produced and harvested from single spore cultures on beads (Pro-Lab Diagnostics Microbank™ Bacterial and Fungal Preservation System) at −80 °C for use in the experiments described herein. We submitted the translation elongation factor 1-α (TEF1-α) sequence of the isolate to NCBI GenBank under accession number MK988559 and the single spore isolated culture to The Agricultural Research Service Collection of Entomopathogenic Fungal Cultures (ARSEF) under the accession number 14325.

To produce inoculum for experiments, we transferred beads from cryovials aseptically and plated them onto 100 mm × 15 mm Petri plates containing dodine-free semi-selective CTC medium [49]. The plates were then incubated at 25 ± 2 °C in the dark for 14–18 days. We harvested the conidia under aseptic conditions by scraping the agar surface with a sterile stainless-steel mini spatula and suspended them in a sterile 0.05% aqueous solution (*v*/*v*) of Triton™ X-100 (Dow Chemical Co., Midland, MI, USA). We homogenized the conidial suspension by vigorously shaking for one minute in sterile capped 50 mL centrifuge tubes. We filtered the conidial suspension through four layers of sterile cheese cloth to separate the mycelial fragments from conidia. We determined the concentration of the stock conidial suspension under a compound microscope at 400× magnification with a Neubauer hemocytometer and adjusted the concentration to 1 × 10^8^ conidia mL^−1^ for seed inoculation. To determine the viability of the conidia we assessed the ability of conidia to form a germ tube by plating 80 μL of the conidial suspension onto a Petri plate (100 mm × 15 mm) containing CTC medium and stored in dark at 25 ± 2 °C for 24 h. We assessed the percent viability by randomly counting 200 conidia at 400× magnification and considered conidia viable if hyphae were visible or the germ tube was at least twice the length of the conidium. The germination rate of the conidial suspensions used in experiments was greater than 90%.

### 2.2. Seed Surface Sterilization and Inoculation

We surface-sterilized seeds of Austrian winter pea (AWP), cereal rye, and canola in a sterile laminar flow hood by immersion in 0.5% sodium hypochlorite for two minutes followed by soaking in 70% ethanol for two minutes and rinsing three times in sterile distilled water [52]. To confirm successful surface sterilization, we placed three randomly selected seeds onto a Petri plate (100 mm × 15 mm) containing CTC medium. We also plated 80 μL of the final rinse water onto 100 mm Petri plates containing Sabouraud dextrose agar and yeast (SDAY) and incubated them in darkness at 25 ± 2 °C for 10 days. After surface sterilization, we air-dried the seeds under sterile conditions in a laminar flow hood for 5 min and stored them at 4 °C for 24 h for use in experiments.

Surface-sterilized seeds were placed in 100 mL of freshly prepared conidial suspension (1 × 10^8^ conidia mL^−1^) of *M. robertsii* in a 250 mL sterile beaker covered with aluminum foil. We placed control seeds in a 250 mL beaker containing 100 mL of 0.05% Triton X-100 aqueous solution and covered it with aluminum foil. We then placed both the inoculated and control beakers on a shaker at 10 rpm for 2 h to accomplish inoculation.

### 2.3. Plant Growth and Experimental Design

We prepared plant growth medium by mixing steamed field soil and potting mix (Vigoro Organic Potting Mix) in a 1:1 ratio by volume. To reduce contaminant microbial growth and interactions, we steamed the growth medium twice for 2 h at 121 °C in a steam sterilizer. We waited 48 h after steaming the medium before using it in experiments to avoid toxicity of the soil to plants. We placed one seed inoculated with *M. robertsii*, or a control seed at a depth of ~2.5 cm in separate steamed 15 cm diameter × 14.7 cm tall plastic plant pots containing the prepared growth medium by using separate sterile spatulas for each treatment group. We planted the control seeds before the *M. robertsii*-inoculated seeds to avoid cross-contamination. We placed the prepared pots randomly on a greenhouse bench with 16L:8D photoperiod at 25 ± 3 °C and provided water as needed. We repeated the experiment three times for AWP and four times for cereal rye and canola. For AWP, in the first, second, and third trial, we grew 10, 16, and 26 *M. robertsii*-inoculated plants, and 10, 15, and 34 control plants, respectively. For cereal rye, in the first, second, third, and fourth trials, we grew 5, 9, 16, and 38 *M. robertsii*-inoculated plants, and 5, 10, 15, and 44 plants control plants, respectively. For canola, in the first, second, third, and fourth trials, we grew 4, 7, 15, and 25 *M. robertsii*-inoculated plants, and 5, 8, 15, and 20 control plants, respectively (Table S1).

### 2.4. Evaluation of Plant Endophytic Colonization

To evaluate the endophytic colonization of plants by *M. robertsii* we sampled treated and control plants at 30 days after germination (DAG). From each plant, we randomly removed a newly emerged true leaf and two 5-cm long root sections using sterile scissors. We wiped the removed leaves with 70% ethanol using Kimwipes™ and rinsed excised roots with tap water to remove loosely attached soil. We stored the excised plant sections individually at 4 °C in 17.7 cm × 18.8 cm labelled Ziploc^®^ bags for further processing. We surface-sterilized the excised leaf and root sections by submerging in 0.5% sodium hypochlorite for three minutes followed by 70% ethanol for two minutes, followed by serially rinsing three times in sterile deionized water. To confirm the effectiveness of tissue sterilization, we plated 80 μL of the final rinse water onto CTC medium and kept the dishes at 25 ± 2 °C for 10 days in darkness. The surface-sterilized leaf and root tissues were dried on a sterile paper towel. We cut off the ~1 mm outer edges of the surface-sterilized leaf and ends of the root tissues using sterile dissecting scissors to remove dead cells. We cut each leaf into 6 mm × 6 mm sections and each root into 6 mm-long sections so that each plant generated six leaf and six root sections. We plated each tissue type from each plant in a labeled Petri dish prepared with CTC medium by pressing the tissue flat against the surface of the medium. The plates were incubated in the dark at 25 ± 2 °C for 14 days to allow endophytic *M. robertsii* growth to emerge from the plant tissue. We identified *M. robertsii* by characteristic white hyphal growth and dark green conidia visually, and under magnification as needed. In our assessment of frequency of tissue colonization, we did not count microbial growth suspected to be other species. To confirm the molecular identity of fungal isolates emerging from the plant sections, we extracted DNA from fungal mycelium produced in 50 mL sterile potato dextrose broth (PDB, 2.4%) [53], and stored at 4 °C for short-term or −20 °C for long-term storage. We performed PCR amplification using forward (EF-1T) and reverse primers (EF-2T) to amplify the intron-rich 5′ portion of translation elongation factor-1 α (5′-TEF) locus [51]. We cleaned the PCR products by digestion with Exo-SapIT^®^ (Affymetrix, Santa Clara, CA, USA) and submitted the products to the Pennsylvania State University Genomics Core Facility for Sanger sequencing. The sequences were compared and analyzed with reference sequences by using the BLASTn tool provided by NCBI. We aligned the sequences using ClustalW and performed phylogenetic analysis using the maximum likelihood method to determine the identity of *Metarhizium* species and we confirmed all the isolates as *M. robertsii*.

For AWP, from the three replicated trials, we plated 354 root and 354 leaf sections from 59 control plants, and we plated 312 root and 312 leaf sections from 52 AWP plants grown from *M. robertsii*-inoculated seeds. For cereal rye plants from four replicated trials, we plated 444 root and 444 leaf sections from 74 control plants, and we plated 408 root and 408 leaf sections from 68 plants grown from *M. robertsii*-inoculated seeds. For canola, from four replicated trials, we plated 288 root sections and 288 leaf sections from 48 control plants and 306 root and 306 leaf sections from 51 *M. robertsii*-inoculated plants (Table S1).

We will refer to control plants as ‘Not Inoculated’ plants because their seeds were not inoculated with *M. robertsii* conidial suspension. *M. robertsii*-inoculated plants from which we recovered or did not recover *M. robertsii* were categorized as ‘Inoculated and Detected’ or ‘Inoculated and Not Detected’, respectively. We considered a plant to be endophytically colonized when we observed growth of *M. robertsii* from one or more root or leaf sections of a plant. We calculated proportion endophytic colonization of plants by dividing total number of plants with root, leaf, or both root and leaf colonization by total number of *M. robertsii*-inoculated plants. We determined the relative proportion endophytic colonization by plant tissue type for each cover crop by dividing the total number of root or leaf tissues which produced *M. robertsii* growth by total number of leaf or root tissues that were plated onto CTC medium.

### 2.5. Plant Response to Inoculation with M. robertsii

We measured endophytic colonization, height, and chlorophyll content of plants in all trials, whereas we measured plant above-ground biomass in the second and third experiment for AWP and third and fourth experiment for cereal rye and canola. For all parameters, we measured the control plants before the *M. robertsii*-inoculated plants to avoid potential cross-contamination. We measured plant height (cm) from the base of the plant to the tip of the longest fully emerged true leaf at 30 DAG. We measured the total chlorophyll content (SPAD units) of the emerged true leaf by using a SPAD-502 Plus Chlorophyll Meter (Konica Minolta, Japan). We measured chlorophyll from three different true leaves of each plant. Between each treatment, the ruler and chlorophyll meter were wiped with 70% ethanol using Kimwipes™ to avoid contamination. We measured plant above-ground biomass by cutting the plant at the soil-plant interface with clean scissors. We placed the plant biomass in dried, pre-weighed brown paper bags, and oven-dried them at 60 °C for 10–14 days, when biomass was weighed using a digital balance.

### 2.6. Statistical Analyses

We performed all analyses in JMP^®^ Pro 13.2.0 (SAS Institute Inc., Cary, NC, USA, 1989–2007) unless otherwise stated. We used mixed model ANOVA to determine the degree of endophytic colonization among cover crop species, degree of endophytic colonization in different plant tissues within and among cover crop species, plant height, chlorophyll content and above-ground biomass. To determine endophytic colonization of cover crop species, we analyzed endophytic colonization for ‘leaf only’, ‘root only’ and ‘both leaf and root’ groups for each cover crop species separately. To determine endophytic colonization for a specific tissue, we analyzed ‘root’ or ‘leaf’ endophytic colonization for each cover crop species separately. We designated all treatment variables as fixed factors and block as a random factor. When the model was significant, we used Tukey’s Honest Significant Difference post-hoc test as pairwise tests of means between each treatment and considered analysis significant at *p* < 0.05. We conducted regression analyses to assess the relationship between the proportion of *M. robertsii*-colonized root and leaf sections per plant and plant growth parameters, using proportion of colonized tissue sections as the explanatory variable and plant growth measures as response variable. For all analyses, we transformed proportions using square root arcsine transformation to meet assumptions of normality, equality of variances and to reduce heterogeneity of variances [54]. Data presented in figures and tables are not transformed.

## 3. Results

### 3.1. Endophytic Colonization of Plants and Tissue Location

We did not detect *M. robertsii* from any of the non-inoculated control plants for any of the experiments (data not shown). Therefore, we consider it most likely that detection of *M. robertsii* in treated plants was due to systemic colonization from the treated seed. We recovered *M. robertsii* from AWP (58.72 ± 0.62%; *n* = 52), cereal rye (46.45 ± 3.91%; *n* = 68), and canola (39.05 ± 3.74%; *n* = 51) plants grown from inoculated seeds. When we compared among cover crop species, we found greater endophytic colonization in AWP than in cereal rye and canola. There was no difference in endophytic colonization between canola and cereal rye (*p* < 0.03, *F*_2,91_ = 3.8, *n* = 94). Therefore, our hypothesis that colonization of AWP would be greater than in cereal rye, and that colonization of canola would be lowest was partially supported.

**Figure 1:**
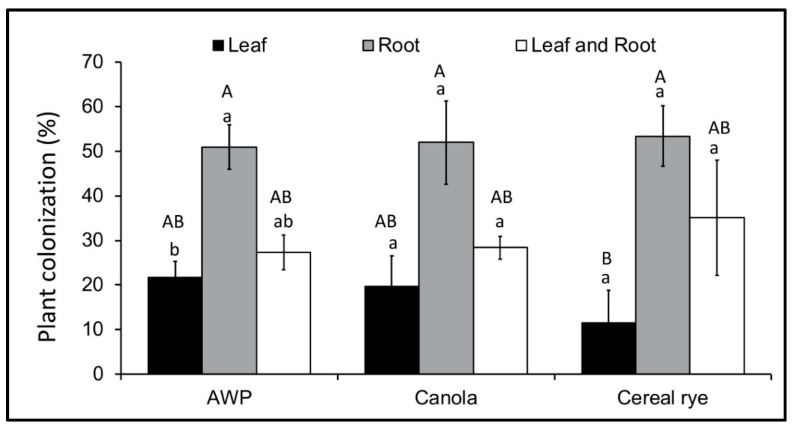
Percentage of plants grown from *M. robertsii*-inoculated seeds from which endophytic growth was detected in only leaf, only root or both leaf and root tissues in Austrian winter pea (AWP) (*n* = 30), cereal rye (*n* = 38) and canola (*n* = 26). The error bars represent ± standard error of the mean. The lowercase letters show the difference for leaf only, root only and both leaf and root within a cover crop species. The uppercase letters show the differences for leaf only, root only and both leaf and root groups among cover crop species. The bars sharing common letters are not significantly different at *p* = 0.05.

Our hypothesis that we would detect colonization more frequently in roots compared to foliage was partially supported. Out of 30 AWP plants in the group ‘Inoculated and Detected’, we detected endophytic colonization only in leaf tissue in 7 plants (21.75 ± 3.54%), only in root tissue in 15 plants (50.95 ± 4.97%), and endophytic colonization of both leaf and root tissues in 8 plants (27.03 ± 3.9%). Out of 38 cereal rye plants in the group ‘Inoculated and Detected’, we detected endophytic colonization only in leaf tissue in 5 plants (11.51 ± 7.3%), only in root tissue in 15 plants (53.41 ± 6.76%), and endophytic colonization of both leaf and root tissues in 18 plants (35.08 ± 12.93%). Out of 26 canola plants in the group ‘Inoculated and Detected’, we detected endophytic colonization only in leaf tissue in 4 plants (19.72 ± 6.8%), only in root tissue in 15 plants (51.95 ± 9.33%), and endophytic colonization both in leaf and root tissues in 7 plants (28.33 ± 2.55%). There was no significant difference among cover crop species in the proportion of plants from which we detected endophytic colonization only in leaf tissue (*p* < 0.63, *F*_2,5_ = 0.52, *n* = 9), only in root tissue (*p* < 0.94, *F*_2,4_ = 0.06, *n* = 9), or both leaf and root tissue (*p* < 0.61, *F*_2,4_ = 0.57, *n* = 9). Among different tissues within a cover crop species, the proportion of endophytic colonization only from root was significantly higher in AWP plants compared to recovery only from leaf tissue, whereas the recovery from both leaf and root tissues was not different than recovery from only leaf or only root tissues (*p* < 0.03, *F*_2,4_ = 9, *n* = 9). The proportion of endophytic colonization among tissues was not different in cereal rye (*p* < 0.15, *F*_2,4_ = 3.19, *n* = 9) or canola (*p* < 0.11, *F*_2,4_ = 3.97, *n* = 9) (Figure 1).

**Figure 2:**
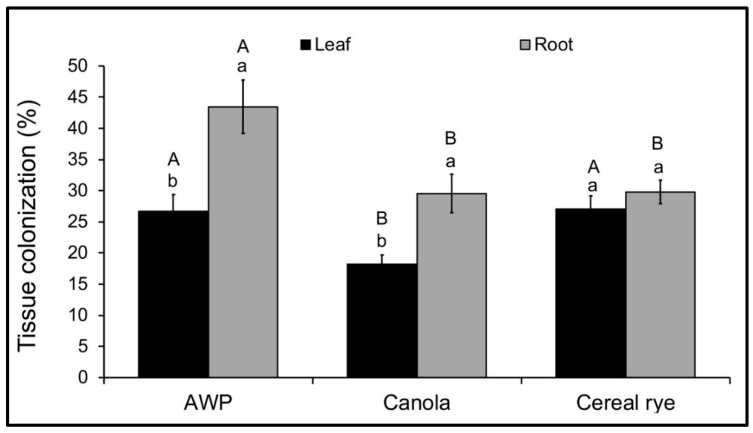
Percentage of root or leaf endophytically colonized in cover crops grown from *M. robertsii*-inoculated seeds of AWP (leaf *n* = 312, root *n* = 312), cereal rye (leaf *n* = 408, root *n* = 408) and canola (leaf *n* = 306, root *n* = 306). Error bars represent ± standard error of the mean. The lowercase letters show the difference between root and leaf colonization within a cover crop species. The uppercase letters show the differences among cover crop species where leaf or root tissues were analyzed separately. The bars sharing common letters are not significantly different at *p* = 0.05.

Within a cover crop species, endophytic colonization of root tissue was greater than colonization of leaf tissue in AWP (*p* = 0.007, *F*_1,28_ = 8.23, *n* = 30) and canola (*p* = 0.02, *F*_1,24_ = 6.72, *n* = 26), and was not different in cereal rye (*p* = 0.43, *F*_1,36_ = 0.64, *n* = 38). Among the cover crop species, endophytic colonization of root tissue was greater in AWP than in cereal rye and canola, while there was no difference between canola and cereal rye (*p* = 0.004, *F*_2,74_ = 5.89, *n* = 78). When compared among cover crop species, endophytic colonization of leaf tissue was greater in AWP and cereal rye than in canola, whereas colonization of leaf tissue of AWP and cereal rye was not different (*p* = 0.03, *F*_2,90_ = 3.81, *n* = 94) (Figure 2).

### 3.2. Plant Growth Response to Seed Inoculation with M. robertsii

**Figure 3:**
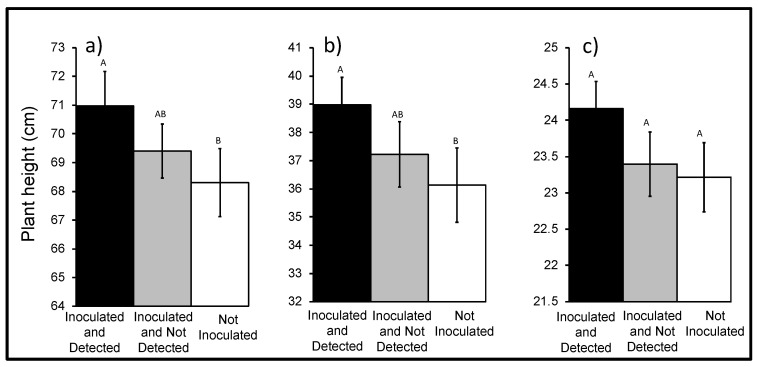
Height of ‘Inoculated and Detected’ or ‘Inoculated and Not Detected’ and ‘Not Inoculated’ (**a**) AWP (*n* = 111), (**b**) cereal rye (*n* = 142), and (**c**) canola (*n* = 99). Error bars represent ± standard error of the mean. The bars sharing common letters are not significantly different at *p* = 0.05.

The height of AWP plants from the ‘Inoculated and Detected’ group (70.98 ± 1.20 cm) was greater than height of the ‘Not Inoculated’ control (68.3 ± 1.18 cm) plants (*p* = 0.036, *F*_2,106_ = 3.44, *n* = 111), but was not significantly different from ‘Inoculated and Not Detected’ plants (69.40 ± 1.63 cm) (Figure 3). The height of ‘Inoculated and Detected’ cereal rye (38.98 ± 0.98 cm) was greater than the height of ‘Not Inoculated’ (36.13 ± 1.32 cm) (*p* = 0.03, *F*_2,137_ = 3.54, *n* = 142) but was not different than the height of ‘Inoculated and Not Detected’ (37.22 ± 1.16 cm) (Figure 3). The height of ‘Inoculated and Detected’ canola plants (24.16 ± 0.38 cm) was not significantly different from height of ‘Not Inoculated’ (23.22 ± 0.48 cm) or ‘Inoculated and Not Detected’ (23.40 ± 0.44 cm) plants (*p* = 0.22, *F*_2,96_ = 1.54, *n* = 99) (Figure 3).

**Figure 4:**
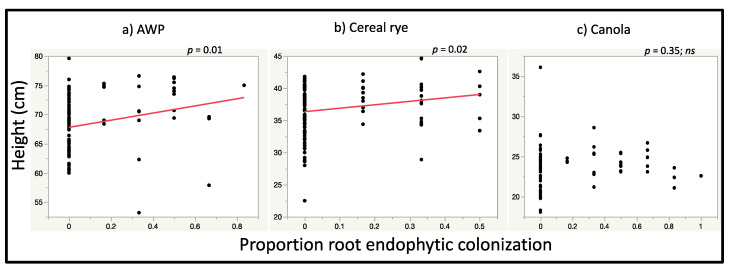
Relationship between the relative proportions of endophytic colonization of root tissue sections from which *M. robertsii* was recovered and height of (**a**) AWP, (**b**) cereal rye and (**c**) canola. No *M. robertsii* was recovered from non-inoculated control leaf and root tissue sections (data not shown). Regression lines for significant relationships between endophytic colonization and plant height are shown in red.

Our hypothesis that the level of growth promoting effects would be associated with intensity of colonization was partially supported. We found a relationship between root, but not foliar colonization, and growth promoting effects. In regression analysis to assess the relationship between plant height with the relative proportion of leaf and root tissues from which we recovered endophytic *M. robertsii*, the height of ‘Inoculated and Detected’ AWP was positively correlated with the proportion of endophytic colonization of root sections (*r^2^_Adj_* = 0.06, *p* = 0.01, estimate = 4.62) but not leaf sections from which *M. robertsii* was recovered (*r^2^_Adj_* = 0.02, *p* = 0.13, estimate = 4.76) (Figure 4). The height of ‘Inoculated and Detected’ cereal rye was positively related to the proportion of root sections from which *M. robertsii* was recovered (*r^2^_Adj_* = 0.04, *p* = 0.02, estimate = 3.49) but not leaf sections (*r^2^_Adj_* = 0.004, *p* = 0.23, estimate = 2.46) (Figure 4). The height of ‘Inoculated and Detected’ canola plants was not correlated with the proportion of root (*r^2^_Adj_* = −0.001, *p* = 0.35, estimate = 0.75) nor leaf sections from which *M. robertsii* was recovered (*r^2^_Adj_* = 0.001, *p* = 0.29, estimate = 2.21) (Figure 4).

**Figure 5:**
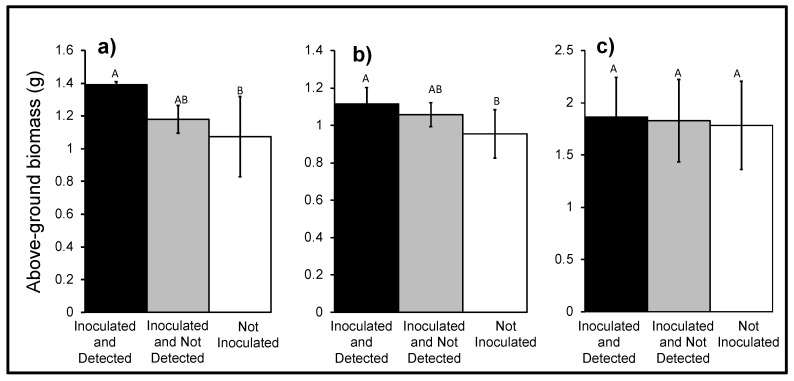
Mean above-ground dry biomass of (**a**) AWP (*n* = 91), (**b**) cereal rye (*n* = 113), and (**c**) canola (*n* = 75) plants. Aboveground biomass was measured at 30 DAG by drying at 60 °C for 10–14 days. Error bars represent ± standard error of the mean. The bars sharing common letters are not significantly different within a cover crop species.

The above-ground dry biomass of ‘Inoculated and Detected’ (1.40 ± 0.02 g) AWP plants was greater than biomass of ‘Not Inoculated’ (1.07 ± 0.25 g) plants (*p* = 0.02, *F*_2,87_ = 3.94, *n* = 91), but was not different from the ‘Inoculated and Not Detected’ (1.18 ± 0.09 g) plants (Figure 5). Above-ground dry biomass of ‘Inoculated and Detected’ (1.11 ± 0.09 g) cereal rye plants was greater than the biomass of ‘Not Inoculated’ (0.95 ± 0.13 g) plants (*p* = 0.04, *F*_2,109_ = 3.12, *n* = 113), but was not different from the ‘Inoculated and Not Detected’ (1.06 ± 0.06 g) plants (Figure 5). The above-ground dry biomass of ‘Inoculated and Detected’ (1.86 ± 0.38 g) canola plants was not different than ‘Not Inoculated’ (1.78 ± 0.42 g) plants and the ‘Inoculated and Not Detected’ (1.83 ± 0.40 g) plants (*p* = 0.40, *F*_2,71_ = 0.92, *n* = 75) (Figure 5).

**Figure 6:**
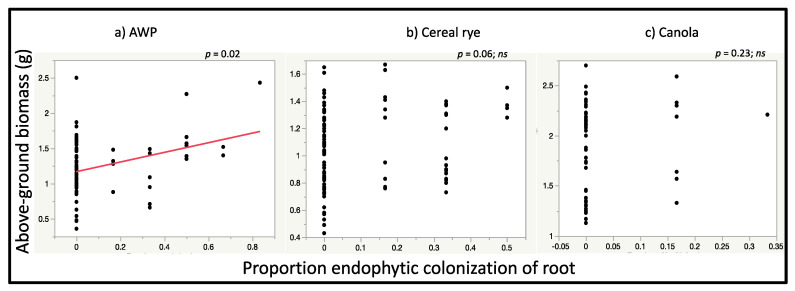
Relationship between the relative proportion of root sections from which *M. robertsii* was recovered and above-ground dry biomass of (**a**) AWP. There was no relationship between proportion of root sections from which *M. robertsii* was recovered and above-ground dry biomass of (**b**) cereal rye, and (**c**) canola. There was no relationship between the relative proportion of leaf sections from which *M. robertsii* was recovered and above-ground dry biomass of any cover crop species (data not shown). Regression lines for significant relationships between endophytic colonization and plant above-ground biomass are shown in red.

In analyses to assess the correlation of above-ground dry biomass of plants with the proportion of leaf and root sections from which *M. robertsii* was recovered, the above-ground biomass of AWP was positively correlated with the proportion of root sections from which *M. robertsii* was recovered (*r^2^_Adj_* = 0.07, *p* = 0.02, estimate = 0.41) but not to the proportion of endophytic leaf sections (*r^2^_Adj_* = 0.03, *p* = 0.09, estimate = 0.49) (Figure 6). The above-ground dry biomass of cereal rye was not correlated with the proportion of leaf (*r^2^_Adj_* = 0.01, *p* = 0.17, estimate = 0.22) or root sections (*r^2^_Adj_* = 0.03, *p* = 0.06, estimate = 0.22) from which *M. robertsii* was recovered (Figure 6). Similarly, above-ground dry biomass of canola plants was not correlated with the proportion of leaf (*r^2^_Adj_* = −0.003, *p* = 0.37, estimate = 0.35) or root sections (*r^2^_Adj_* = 0.008, *p* = 0.23, estimate = 0.19) from which *M. robertsii* was recovered (Figure 6).

The chlorophyll content of AWP (*p* = 0.3, *F*_2,106_ = 1.22, *n* = 111), cereal rye (*p* = 0.6, *F*_2,137_ = 1.22, *n* = 142), and canola (*p* = 0.6, *F*_2,96_ = 0.52, *n* = 99) in ‘Inoculated and Detected’ plants was not significantly different from ‘Not Inoculated’ plants or ‘Inoculated and Not Detected’ plants (data not shown). Chlorophyll content of AWP, cereal rye, and canola plants grown from *M. robertsii*-inoculated seeds was not related with the relative proportion of leaf or root sections from which *M. robertsii* was recovered (data not shown).

## 4. Discussion

Interest in the use of soil microbes in agroecosystems to promote plant growth and manage insect pests has seen rapid growth over the last decade [55,56]. Similarly, research on the effects of endophytic entomopathogenic fungi, including *Metarhizium* spp., on plant performance has become a focus of extensive research since the discovery of their relationship with and beneficial effects on plants [1,57].

We inoculated seeds of winter cover crop species in different plant families with spores of *M. robertsii* to establish endophytic colonization. When colonization of leaf and root tissues were compared within a cover crop species, we found greater colonization of root tissue compared with leaf tissue of Austrian winter pea (AWP) and canola but no difference between root and leaf colonization of cereal rye. For AWP and canola, but not cereal rye, these results partially support our hypothesis that we would detect *M. robertsii* more frequently in root than in leaf tissues. When compared among cover crop species, root colonization in AWP was greater than in cereal rye or canola. Similarly, leaf colonization was greater in AWP and cereal rye than in canola. When we compared overall endophytic colonization among cover crop species, we found greater endophytic colonization in AWP than cereal rye and canola and there was no difference between canola and cereal rye. These results are partially consistent with our previous research in which detection of *M. robertsii* from field soil in which AWP was grown as a cover crop was greater than those in which canola was grown or where cereal rye predominated in cover crop mixtures [44]. Therefore, our hypothesis that overall colonization of AWP would be greater than in cereal rye, and that colonization of canola would be lowest was partially supported. These differences from field observations may be due to the absence of other belowground biotic interactions or environmental conditions that differed between the relatively controlled conditions in our experiment compared with those interactions and conditions in our field experiment. Our results are consistent with previous reports in which *Metarhizium* spp. endophytically colonized diverse plant species grown from seed, soil or foliar inoculation. For example, successful endophytic colonization by seed inoculation with *M. robertsii* and *M. acridum* was achieved in cowpea and cucumber [58]. *M. anisopliae* endophytically colonized 82.6% of broad bean plants at 30 days after seed inoculation and 7 days after soil inoculation [16]. Foliar inoculation with *M. anisopliae* resulted in endophytic colonization of 80% of 4-week old canola plants [11]. Inoculation with *M. robertsii* or *M. anisopliae* established endophytic colonization in tomato [18,59], sorghum [60], tea [61], soybean [23], switchgrass and haricot bean root [6], cauliflower root [62] and cassava root [19]. It appears that *M. robertsii* is a generalist endophyte capable of establishing varying degrees of endophytic colonization in multiple tissues across a broad plant host range that includes many plant families and functional traits.

Our hypothesis that we would detect colonization more frequently in roots compared to foliage was partially supported. The proportion of plants from which we detected only root colonization was not different among the three cover crop species, and detection of endophytic colonization only in roots was greater than colonization only in leaf tissue. However, there was no difference in the proportion of plants in which we detected colonization of only root tissue and of both root and leaf sections. This suggests that some plants for which we detected *M. robertsii* colonization only in root or only leaf tissue may have a localized or transient endophytic colonization that may change over time. Similarly, the plants from which we detected endophytic colonization in both root and leaf tissue may have hosted systemic endophytic colonization [62,63]. Because we did not detect *M. robertsii* in any of the control plants, which were randomly located among treated plants, we consider the possibility of foliar infection through horizontal transmission or cross-contamination unlikely.

The method of inoculation may also contribute variability to endophytic colonization. Our study is consistent with previous reports in which *Metarhizium* spp. colonized plant tissues grown from inoculated seeds. For example, *M. anisopliae* seed inoculation of *Vicia faba*, a legume, resulted in endophytic colonization of root tissue at 30 days after inoculation of seed and 7 days after inoculation of soil [16]. The authors only evaluated root tissue of inoculated plants and reported that the degree of colonization in roots differed among tested fungal isolates. Cowpea and cucumber plants grown from seeds inoculated with different *M. robertsii* and *M. acridum* isolates showed endophytic colonization of both leaf and root tissue [58]. In our study, the method of establishing endophytic *M. robertsii* by seed inoculation may have supported greater root colonization compared with leaf colonization. Different methods of inoculation have supported establishment of a variable degree of endophytic colonization in above- and below-ground plant tissues [64]. Foliar and soil application of *M. robertsii* or *M. anisopliae* established endophytic colonization in tomato leaf, stem and root [18,59], sorghum leaf and stem [60], tea leaf and root [61], soybean leaf, stem and root [23], switchgrass and haricot bean root [6], cauliflower root [62] and cassava root [19]. Foliar application of *M. anisopliae* established up to 80% endophytic colonization of above-ground tissue in 4-week old canola [11], which is greater than the level of colonization that we observed in leaf tissue by seed inoculation of canola. Foliar application [11] may have resulted in greater endophytic colonization compared with greater root colonization that we observed in our study. Inoculation of tea plants by foliar spray with *M. anisopliae* favored leaf colonization whereas soil inoculation favored root colonization [61]. *M. robertsii* and *M. anisopliae* colonized soybean plants systemically following leaf and root immersion, resulting in greater root colonization by root immersion and greater leaf colonization by leaf immersion, but detection of endophytic colonization declined over time [23]. As a predominantly soil-dwelling fungus, we expect that in nature root colonization by *Metarhizium* spp. would be more common than colonization of other tissues if colonization is mainly localized, but that other tissues could be colonized through systemic colonization.

The degree of endophytic colonization and specific tissue harboring endophytes also varied among different studies. Our results contrast with reports that *Metarhizium* is exclusively an endophyte of roots [17,20,22,62]. We detected *M. robertsii* more frequently in root tissue than in leaf tissue in all plants, but colonization of both root and leaf tissue was common. These results are consistent with studies that report *M. robertsii* or *M. anisopliae* as an endophyte of stems and leaves as well as roots [11,18,58,59,60,61]. Greater colonization and frequency of detection in plant organs near to the inoculation site may help explain greater detection in roots than in leaves [19]. Genotype-specific interactions between endophytes and their host can greatly influence the degree and persistence of endophytic relationship [65]. Increasing our understanding of the factors that control the location of endophytic growth may contribute to the optimization of establishing endophytic growth to exploit the benefits of *Metarhizium* spp. to plants.

Our hypothesis that the level of growth promoting effects would be associated with intensity of colonization was partially supported. The demonstration in this and other studies of the plant growth-promoting effects of *Metarhizium* spp. suggests a possible mechanism of growth promotion action [4,22,25,66,67]. For example, seed inoculation followed by endophytic root colonization with *Metarhizium* spp. increased stalk length, ear and foliage biomass of maize, and increased plant height [10,18,59], and maize seeds inoculated with *M. anisopliae* under field conditions showed an increased stand density, and stalk and foliar fresh weight [29]. The positive effect of endophytic colonization on AWP and cereal rye height and above-ground biomass in our study may be due in part to the contribution of *M. robertsii* to improved nutrient acquisition and assimilation, or production of plant growth promoting auxins [5,6,24,27]. As described for *Trichoderma* spp., plant growth promotion by *Metarhizium* spp. may be multifactorial [68]. Endophytic *M. robertsii* did not affect the height and above-ground biomass of canola plants in our study. These results are consistent with previous studies on the effect of brassicas and legumes on soil microbes [69] where plant root exudates and secondary metabolites regulate the fungal rhizosphere community [33,70]. The lack of growth effects on canola and less frequent tissue specific detection of *M. robertsii* in our study could be due to the adverse effects of glucosinolates or other compounds associated with brassicaceous plants [71]. No effect of *M. robertsii* colonization on growth of canola and positive effects on AWP and cereal rye suggests host-dependent variability of interactions. Plant-specific interactions between endophytes and their host can affect the degree and persistence of endophytic relationship and the subsequent effects on plant growth parameters [65,68].

We did not observe any effect of endophytic colonization on the chlorophyll content of any cover crop species. These results are consistent with our previous study [10] and other studies that report a neutral or negative effect of endophytic colonization on chlorophyll content. For example, in a field experiment, inoculation of maize seeds with *M. robertsii*, *M. brunneum* and *M. anisopliae* did not affect chlorophyll content [22]. Another study involving foliar application of *M. robertsii* to sorghum and subsequent re-isolation reported no effect on chlorophyll content or photosynthetic activity [60].

In our study, the regression analysis indicated a weak and partial dose-dependent effect on plant growth promotion in which plant height and above-ground biomass were positively but weakly correlated with the proportion of root, but not leaf, tissues from which endophytic *M. robertsii* was recovered in AWP, but not cereal rye or canola. These results indicate that higher rates of root colonization may produce greater benefits in some plant species. More intensive endophytic colonization of plants by increasing the inoculum concentration may provide a more pronounced effect on the growth of plants and their ability to withstand stress from herbivory or other environmental factors. Similar studies have explored concentration-dependent responses of endophytic colonization of *Metarhizium* spp. in host plants. Ahmad et al. [10] observed colonization intensity-dependent effects of *M. robertsii* that resulted in a positive relationship between number of tissue sections endophytically colonized and growth promotion of maize and a negative relationship with growth rate of the black cutworm, *Agrotis ipsilon*, feeding on endophytic maize foliage. Soil inoculation with *M. brunneum* showed reduced Fe chlorosis in sorghum plants grown in the calcareous soil inoculated with a higher dose of 5 × 10^8^ conidia mL^−1^, and the two highest doses (5 × 10^6^ and 5 × 10^8^ conidia mL^−1^) increased plant height and inflorescence production of sunflower grown in both soils as compared to lower doses [72]. Seed inoculation of broad bean with *M. brunneum* for 16 h resulted in relatively higher root colonization and greater plant growth than those plants from seeds inoculated for 2 h or 8 h [66]. Those authors reported inconsistent effects on all plant growth parameters measured among inoculation treatments, and they suggested that increasing the seed inoculation duration may provide more time for a greater intensity of endophytic colonization. The duration of seed inoculation, effective fungal spore concentration, the method of inoculation, plant species, isolate diversity and time interval between inoculation and evaluation may be the factors affecting that contribute to the intensity and location of endophytic colonization, and in the varying degree of effects on host plants and insect pests. Because we analyzed only a fraction of each plant to assess the intensity of endophytic colonization, we measured relative levels of colonization. Further research is needed to be able to better assess the level of colonization and if there is an intensity of colonization that may result in negative effects on plants.

## 5. Conclusions

Through seed inoculation, we successfully established systemic colonization of endophytic *M. robertsii* in three cover crop species that resulted in plant growth-promoting effects. Several challenges remain before this greenhouse-based information can be transferred to the field. For conservation biological control, it would be useful to know what factors influence the establishment of naturally-occurring systemic and localized endophytic colonization in plants. Soil microbial diversity is positively influenced by crop rotation and diversity [73] and adding cover crops to a rotation that can readily host endophytic colonization may help to conserve or increase beneficial microbes, like *Metarhizium* spp., in the soil microbial community. We suggest that cover crops can help to conserve *Metarhizium* population between cash crop growing seasons, and perhaps benefit endophytic colonization of cash crops and biological control of pests, but this remains to be tested in the field.

## Supplementary Materials

The following are available online at https://www.mdpi.com/2309-608X/6/2/64/s1, Table S1: Supplementary data on endophytic colonization.
