# Supplementary material for: Systemic Colonization by Metarhizium robertsii Enhances Cover Crop Growth
Source: J Fungi (Basel). 2020 May 17;6(2):64. doi: 10.3390/jof6020064 (PMC7344985; doi:10.3390/jof6020064)
Supplement: Supplementary file 1 [file jof-06-00064-s001.pdf]

Table S1. Number of control and treated plants, and number of treated plants from which *M. robertsii* was recovered or not recovered 14-18 days after plating on CTC medium.

Evaluation	Austrian winter pea				Cereal rye					Canola				
	**Trial 1**	**Trial 2**	**Trial 3**	**All trials**	**Trial 1**	**Trial 2**	**Trial 3**	**Trial 4**	**All trials**	**Trial 1**	**Trial 2**	**Trial 3**	**Trial 4**	**All trials**
No. control plants	10	15	34	59	5	10	15	44	74	5	8	15	20	48
No. inoculated plants	10	16	26	52	5	9	16	38	68	4	7	15	25	51
No. inoculated plants with *M. robertsii* detection	6	10	14	30	0	5	12	21	38	0	3	8	15	26
No. leaf sections plated from inoculated plants	60	96	156	312	30	54	96	228	408	24	42	90	150	306
No. leaf sections from inoculated plants with *M. robertsii* detection	4	5	15	24	0	4	9	13	26	0	2	3	7	12
No. root sections plated from inoculated plants	60	96	156	312	30	54	96	228	408	24	42	90	150	306
No. root sections from inoculated plants with *M. robertsii* detection	13	24	23	60	0	10	16	33	59	0	3	13	23	39
No. plants with detection only from leaf	1	2	4	7	0	0	3	2	5	0	1	1	2	4
No. plants with detection only from root	3	6	6	15	0	2	7	6	15	0	1	5	9	15
No. plants with detection from both leaf and root	2	2	4	8	0	3	2	13	18	0	1	2	4	7
